# Supplementary material for: Optimization of the Extraction of Antioxidant Compounds from Roselle Hibiscus Calyxes (Hibiscus sabdariffa), as a Source of Nutraceutical Beverages
Source: Molecules. 2023 Mar 14;28(6):2628. doi: 10.3390/molecules28062628 (PMC10051257; doi:10.3390/molecules28062628)
Supplement: Supplementary file 1 [file molecules-28-02628-s001.zip › molecules-2267480-supplementary.pdf]

Supplementary Materials

Optimization of the Extraction of Antioxidant Compounds from Roselle Hibiscus Calyxes (*Hibiscus sabdariffa*), as a Source of Nutraceutical Beverages

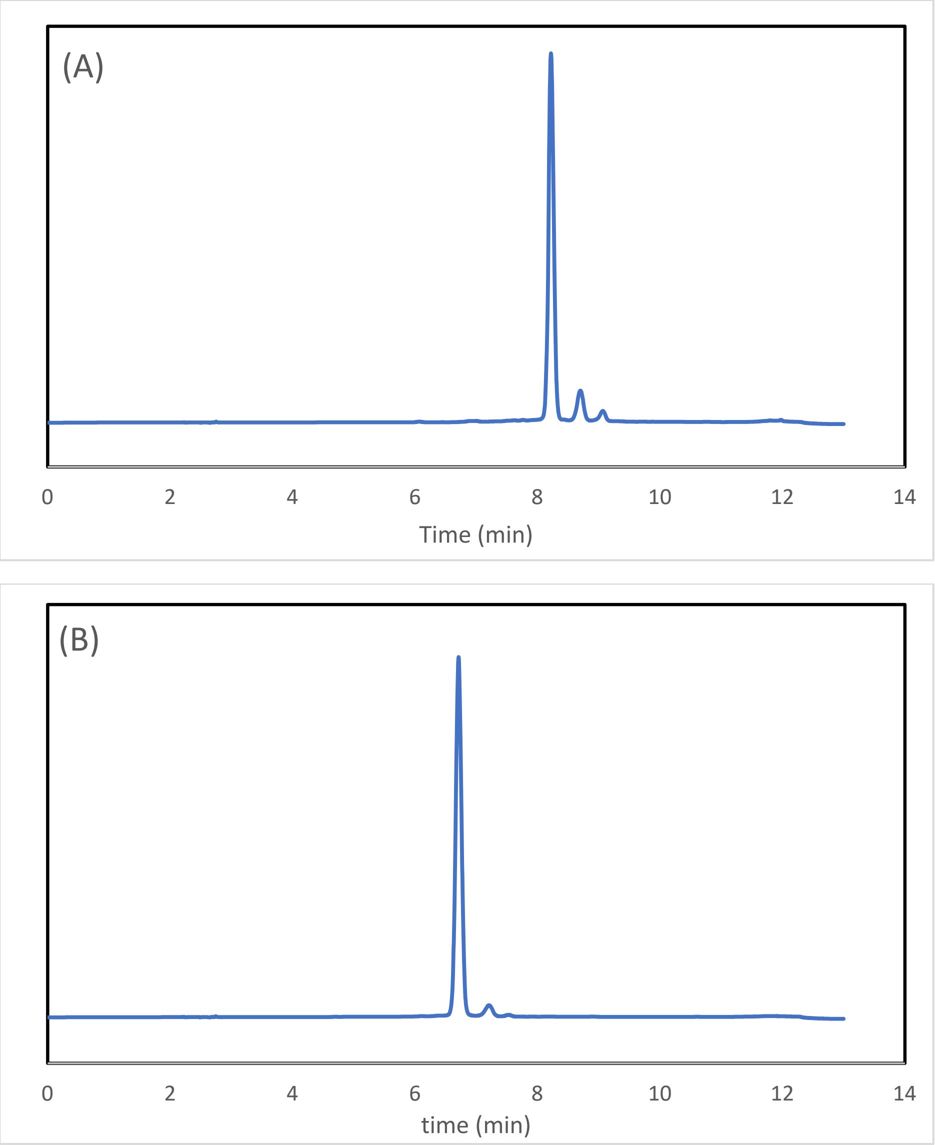

Figure S1. HPLC Chromatographs for anthocyanins standards at 530 nm. (A) cyanidin-3-O-sambubioside. (B) delphinidin-3-O-sambubioside

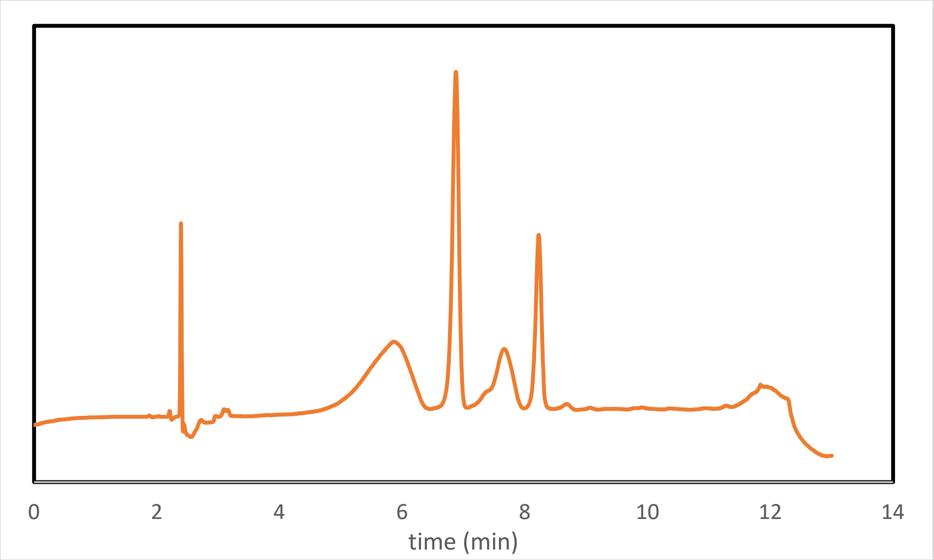

Figure S2. HPLC Chromatographs for a hibiscus sample at 530 nm.

Table S1. Grouping letter for tuckey test of Total Phenolic content (TPC) with 95% confidence level.

Experiment	N	Average	Grouping
6	2	22,96	A
5	2	22,090	A B
15	2	20,9856	A B C
18	2	20,778	A B C
17	2	20,59	A B C D
22	2	19,658	A B C D
8	4	19,085	A B C D
27	2	18,71	A B C D E
25	2	18,38	A B C D E
7	2	18,24	A B C D E
16	2	17,569	A B C D E F
20	2	17,22	A B C D E F
3	2	16,38	A B C D E F
23	2	16,305	A B C D E F
21	2	15,888	A B C D E F G
14	2	15,834	A B C D E F G
12	2	15,533	A B C D E F G H
19	2	14,29	B C D E F G H
1	2	14,11	B C D E F G H
10	2	13,69	C D E F G H I
4	2	12,3819	D E F G H I
11	2	10,61	E F G H I
26	2	9,2824	F G H I
13	2	7,861	G H I
24	2	7,451	H I
2	2	5,580	I

Table S2. Grouping letter for tuckey test of Proanthocyanidin Compounds (PAC) with 95% confidence level.

Experiment	N	Average	Grouping
6	2	3,32	A
5	2	3,00	A
20	2	2,88	A
18	2	2,68	A
27	2	2,67	A
15	2	2,58	A
17	2	2,54	A
8	4	2,522	A
7	2	2,39	A
16	2	2,39	A
19	2	2,299	A
22	2	2,21	A
25	2	2,14	A
14	2	1,968	A
21	2	1,916	A
23	2	1,910	A
12	2	1,897	A
1	2	1,614	A
10	2	1,614	A
4	2	1,096	A
11	2	0,899	A
26	2	0,731	A
3	2	0,656	A
13	2	0,586	A
24	2	0,472	A
2	2	0,3324	A
